# Prognostic values of modifiable risk factors for cardiovascular events in South African health promotion

**DOI:** 10.1371/journal.pone.0271169

**Published:** 2022-08-10

**Authors:** Jacobeth T. Kganakga, Petra Bester, Cristian Ricci, Shani Botha-Le Roux, Marike Cockeran, Minrie Greeff, Iolanthé M. Kruger

**Affiliations:** 1 Africa Unit for Transdisciplinary Health Reseach (AUTHeR), North-West University, Potchefstroom, South Africa; 2 MRC Unit on Hypertension and Cardiovascular Disease, Hypertension in Africa Research Team (HART), North-West University, Potchefstroom, South Africa; 3 School for Computer, Statistical and Mathematical Sciences, Faculty of Natural and Agricultural Sciences, North-West University, Potchefstroom, South Africa; UT Southwestern: The University of Texas Southwestern Medical Center, UNITED STATES

## Abstract

**Background:**

Cardiovascular diseases (CVDs) are increasing at an alarming rate among the South African population. This study aimed to determine the prognostic value of modifiable CVD risk factors for fatal and non-fatal events to inform cardiovascular health promotion practices in the South African public health system.

**Methods:**

Data was collected from individuals participating in the South African leg of a multi-national prospective cohort study. Binary logistic regression was applied to estimate odds of total, non-fatal and fatal cardiovascular events.

**Results:**

Binary logistic regression analyses identified age as a predictor of non-fatal and fatal CV events, with ORs of 1.87 to 3.21, respectively. Hypertension increased the odd of suffering a non-fatal CV event by almost two and a half (OR = 2.47; 95% CI = 1.26, 4.85). Moreover, being physically active reduced the odd of non-fatal CVD events by 38% (OR = 0.62; 95% CI = 0.46, 0.83 for 1 Standard deviation increase of the weighted physical activity index score (WPA)). On the one hand, gamma-glutamyltransferase (GGT) was associated with a higher fatal cardiovascular disease risk OR = 2.45 (95% CI = 1.36, 4.42) for a standard deviation increase.

**Conclusions:**

Elevated blood pressure, GGT, and physical activity have significant prognostic values for fatal or non-fatal CV events. These findings emphasise the importance of highlighting hypertension and physical activity when planning cardiovascular health education and intervention programmes for this population, with attention to the monitoring of GGT.

## Introduction

Incidence and prevalence rates of cardiovascular diseases (CVDs) have been on the rise in Sub-Saharan African countries, including South Africa, since the 1990s [1]. Researchers have since turned the spotlight towards CVD risk factors and prevalence in the African context, especially in black populations [2]. Most studies have been conducted in high-income countries, which differ from the African context in terms of social macro-environments [3, 4], health care systems, access to health care [5] and rates of epidemiological transition [6, 7].

The epidemiological transition has led to a health transition, rural-urban migration and improved living conditions in some rural parts of Africa, including South Africa [8, 9]. Nevertheless, in South Africa, cardiovascular (CV) events such as coronary heart disease (CHD), hypertension, stroke, angina, myocardial infarction and heart failure are steadily emerging as the leading causes of mortality in the working group and older populations [10]. A few decades ago, CVDs were not as prevalent amongst the black South African population as they are today [4, 11]. Possible reasons for the noticeable increase in CVDs might include that most black populations i) lived in rural areas, following a traditional prudent diet, rich in high-density lipoproteins (HDL), serving as protection against CHD development [12], ii) had limited technology and were physically very active (i.e. walking distances, fetching wood, water and other resources) [13], iii) had a short life expectancy due to communicable diseases [14] and malnutrition [15, 16] being the common causes of death, poverty and scarcity of health facilities [13]. Therefore, it is crucial to research the prevalence and incidences of CVDs and their associated modifiable risk factors within the black populations in the African context [4, 17, 18].

According to the WHO’s 2018 country profiles report, non-communicable diseases accounted for 51% of all South African deaths [19]. This number exceeds the 2019 HIV disease burden in South Africa [20]. The country’s quadruple disease burden (non-communicable diseases [NCDs] including HIV and tuberculosis; communicable diseases; conditions related to poverty, violence and injuries; and diseases related to mothers and children) in addition to a dichotomous health system imply overburdened public healthcare and costly private healthcare. South African public health systems have responded to the disease burden on multiple levels through re-engineered primary healthcare (PHC) and related services. The South African health promotion policy and strategy (2015–2019) calls for targeted primary, secondary and tertiary prevention, yet cardiovascular health promotion is not a clear priority [21].

The Prospective Urban and Rural Epidemiology (PURE) study became entrenched into the CVD profile of participants residing within a rural cohort and urban cohort in the North West province of South Africa [22]. PURE is an international longitudinal epidemiology consortium of 27 countries, exploring the demographic and epidemiological transitions related to CVDs in urban and rural communities [22]. Data collected over 16 years enabled a comprehensive perspective of the CVD profile within these communities [23–29]. Because the PURE-SA study paralleled government initiatives to address CVD, the research team learned of participants’ continuous uncontrolled hypertension status during data collection, a rise in CVD incidents, and the need for targeted cardiovascular health promotion interventions [30]. This research aimed to identify the five-year prognostic value of modifiable risk factors for fatal and non-fatal CV events within a selected group of South Africans residing in the Dr Kenneth Kaunda District, the urban cohort of the PURE-SA study. The aim was to inform targeted cardiovascular health promotion interventions to strengthen South Africa’s public health system.

## Methods

### Study design and population

The study protocol complied with the Declaration of Helsinki (as revised in 2004) and was approved (04M10 and NWU-00016-10-A1) by the Health Research Ethics Committee of the North-West University, South Africa. Written informed consent was obtained. Participation was voluntarily and participants could withdraw from the study at any time. Participants were informed that all personal information related to this research will be shared with a third party: Population Health Research Institute, McMaster University, Canada. The bio-statistician was granted access to a de-identified data set for performing all statistical analyses. Reporting of the study conforms to the STROBE statement and references to STROBE [31] and the broader EQUATOR guidelines [32].

The multi-national PURE study is a longitudinal study that aims to examine the relationship of societal influences on human lifestyle behaviours, CV risk factors and incidence of chronic NCDs [22]. The current study is part of PURE’s South African leg (PURE-SA), using pre-collected data. A quantitative research approach was followed, pursuing non-experimental research design. Baseline data were collected in 2005 and follow-up data in 2010. Relevant data were investigated retrospectively by identifying associations between exposure (modifiable CV risk factors) and outcomes (fatal and non-fatal CV events) over five years. Since this study aimed to identify the five-year prognostic value of modifiable risk factors for fatal and non-fatal CV events, the analyses included only participants who suffered a CV event(s) 5 years after collecting baseline data. Data from 746 men and 1,263 women, aged 29 to 94 years, were included for analyses.

### Questionnaires

Trained, Setswana-speaking African fieldworkers, emic to the cohorts, conducted the interviews. Standardised semi-structured questionnaires (S1 File) were used to collect socio-demographic data regarding participants’ educational level, smoking and alcohol consumption habits. In addition, information regarding participants’ physical activity level was collected with the validated International Physical Activity Questionnaire (BAECKE) [33].that was modified and validated for this population [34] (S2 File).

### Binary outcome variables

Participants were asked whether they had a medical diagnosis of CVD (angina pectoris, CHD, stroke, myocardial infarction, or heart failure), whether they were receiving any CVD medication, and recorded all their medications. CVDs were self-reported and verified with medical records (clinic cards). Qualified researchers assisted by trained health care workers performed verbal autopsies for all participants who had suffered a fatal CV event. A medical doctor received all the reported events and supporting documents for adjudication and verification of each case and assigned ICD-10 codes.

### Anthropometrical measurements

Anthropometrical measurements were performed at baseline and followed-up according to the standardised methods prescribed by the International Society of Advancement of Kinanthropometry [35]. Height was measured to the nearest 0.1 cm using a stadiometer (Leicester height measure, Seca, Birmingham, UK). Weight was recorded on a portable electronic scale (Precision Health Scale, A & D Company, Japan) to the nearest 0.01 kg with participants in light underwear and shoes removed. Waist- and hip circumferences were measured with a steel tape (Lufkin, Cooper Tools, Apex NC, USA) and recorded to the nearest 0.1 cm. Body mass index (BMI) was calculated by dividing weight in kilograms by height in meters squared and classified using the WHO categories [36] of BMI of < 18.5 kg/m^2^ as underweight, 18.5–24.99 kg/m^2^ as healthy weight, 25–29.99 kg/m^2^ as overweight, and ≥ 30 kg/m^2^ as obese.

### Cardiovascular measurements

After a 10-minute rest period, brachial blood pressure measurements were performed in duplicate (5 minutes apart) on the right upper arm, with a suitable size cuff. The participant was seated upright with the right arm supported at heart level. Systolic blood pressure (SBP), diastolic blood pressure (DBP) and heart rate were measured with a validated OMRON HEM-757 device (Omron Healthcare, Kyoto, Japan).

### Biochemical analyses

Serum and plasma samples were collected and prepared according to standard protocol. Plasma samples were stored on ice until processing, while serum samples were allowed to clot at room temperature for 30 minutes. All samples were stored at –80°C in cryotubes until further processing.

Serum lipids and gamma-glutamyltransferase (GGT) were analysed with a Sequential Multiple Analyser Computer (SMAC), using the Konelab analyser (Thermo Fisher Scientific Oy, Vantaa, Finland). Low-density lipoprotein (LDL) cholesterol was calculated using the Friedewald formula [37]. Plasma glucose was measured with a hexokinase method using the Vitros DT6011 Chemistry Analyser (Ortho-Clinical Diagnostics, Rochester, New York, USA) and reagents. Glycated haemoglobin was determined from whole blood (EDTA) samples, based on ion-exchange high-performance liquid chromatography, with the D-10 Haemoglobin testing system from Bio-Rad (Bio-Rad Laboratories Ltd., Hercules, CA, USA, #220–0101).

### HIV testing

Written informed consent was obtained from each participant individually after pre-counselling was complete. Participants were given a choice to proceed with the testing. Participants’ HIV status was determined from whole blood using the First Response rapid HIV card test (PMC Medical, India). This test was performed according to the protocol of the National Department of Health of South Africa. Any positive First Response test results were confirmed with the Pareeshak card test (BHAT Bio-tech India). Feedback about the results was provided by two trained counsellors during individual sessions shortly before the participants were transported back to their homes. Infected participants were referred to their local clinic or hospital for follow-up and determination of CD4 cell counts.

### Framingham risk score

The Framingham risk score (FRS) was first developed based on the Framingham Heart Study data. It estimates the risk of developing CHD over ten years or more [38]. The FRS uses major risk factor measurements, including age, sex, diabetes, LDL and HDL cholesterol, SBP and smoking, to calculate a score indicative of an individual’s potential future CHD outcome risk [38, 39]. This score is useful for both the individual patient and the clinician to decide whether lifestyle modification and preventive medical treatment are necessary [38, 40]. Ten-year CHD risk can be presented in three categories based on the calculated results, i.e., ≤ 10% is regarded as low risk; 10–20% as an intermediate risk; and > 20% as high risk [41].

### Statistical analyses

Frequencies (N) were reported as percentage (%) values, while arithmetic means, medians, interquartile range (IQR), standard deviations (SD) and 95% confidence intervals (CI) were used to summarise the data. Either parametric statistical analyses or non-parametric statistical analyses were used depending on data distribution. Data are presented as median and interquartile ranges (IQR) [Q1–Q3]. Note that IQR is reported as a range Q1– Q3. One-way analysis of variance (ANOVA) was performed to compare means. Two-sample *z*-tests were performed to compare proportions. To enhance normality and to rescale to a standardised normal distribution, all continuous variables were transformed using Blom’s inverse rank transformation [42]. Notably, when applied to variables included in further logistic regression, this will correspond to an odd for 1 Standard deviation increase.

Point-biserial correlations along with 95% confidence limits performed by Fisher’s transformation were used to explore associations between risk factors (continuous data) and CV event outcomes (binary outcomes). Binomial multivariate logistic regression analyses were used to determine associations between binary outcomes (CV event outcomes) and categorical risk factors. Multicollinearity among factors under investigation was evaluated using visual inspection of heatmaps and variable clustering. Aggregation between strongly collinear variables was performed to avoid multicollinearity. The least absolute shrinkage and selection operator (LASSO regression) was the chosen strategy to define variables for inclusion in the model. Briefly, based on the glmnet package of the R software (vers. 3.1.6), we performed a LASSO regression in which the optimal tuning parameter (Lambda) was chosen after model cross-validation using a random training dataset made by half of the observation. Afterwards, the model selected by LASSO was also investigated through sequential modelling, adding variables to the model unless stability of estimates. This evaluation was performed, adding variables hierarchically from demographic factors to known risk determinants of CVD (biochemical profile, hypertension, Framingham risk score, etc.).

The Receiver Operating Characteristic (ROC) curves were assessed to analyse the prognostic value and model fitting of independent predictors on CV event outcomes for nested models. In addition, the ordinary odds ratio with 95% confidence limits was reported. Finally, the ROC curves on the sequential nested model were reported. Briefly, we started from a minimally adjusted model (age sex, education, locality) and considered an intermediate model with lifestyle variables (WHO BMI categories, hypertension, nested smoking and alcohol use, weighted physical activity index and Framingham risk score) and a fully adjusted model also considering the biochemical profile (gamma-glutamyltransferase, fasting glucose, glycated haemoglobin, total cholesterol, and total triglycerides). All statistical tests were two-tailed, and the type-I error rate was set to 5% (α = 0.05). Unless specified otherwise, all analyses were performed using SAS version 9.4 (SAS Institute, Cary (NC), USA).

### Power calculations

A power calculation based on performances of the binomial logistic model was applied based on the wp.logistic function of the WebPower package, using R software vers 3.6.2. Briefly, considering the number of fatal and non-fatal outcomes, we computed statistical power considering the proportion of outcomes in exposed and non-exposed subjects, where exposure was defined as a binomial variable. Statistical power above 80% or false positive rate below 20% (β < 0.2) were considered meritorious. According to this evaluation, we considered the analysis of non-fatal outcomes as sufficiently powered. According to our evaluations, the analyses of fatal outcomes could be affected by false-negative results.

## Results

Table 1 displays the baseline characteristics of the participants (N = 1,918). Participants who suffered fatal CV events (N = 25) had a median age of 60.43 (50.34–65.90) years old, while those who suffered non-fatal CV events (N = 69 were 57.12 (48.93–65.68) years old. Those who had no CV events were 47.81 (41.62–55.73) years old (p < 0.001). Of the participants who lived in rural areas, 56.0% suffered fatal CV events compared to 34.8% who suffered non-fatal CV events (p = 0.010). Those who had suffered a fatal CV event were more likely to have no formal education (64%) than those who reported non-fatal CV events (~30%). Noteworthy is that participants who had reported non-fatal CV events had a more detrimental cardiovascular profile. Their measurements for SBP, DBP, mean arterial pressure (MAP), and FRS were higher compared to those who had suffered fatal events, even though the use of blood pressure-lowering medication was higher within the non-fatal group (p = 0.002). Furthermore, the non-fatal group reported the lowest weighted physical activity index (p = 0.001) and the highest diabetic prevalence (9.7%) (both p < 0.001). The highest HIV-positive status (16.6%) was reported in participants with no CV events, followed by those who suffered non-fatal CV events (11.6%) and then fatal CV events (4.2%). No differences were observed in lipid and body composition measures between the groups.

**Table 1:** Baseline demographic characteristics of study participants.

		No CVD outcomes N = 1,918	Non-fatal CVD outcomes N = 69	Fatal CVD outcomes N = 25	Pvalue
	Age	47.81(41.62–55.73)	57.12(48.93–65.68)	60.43(50.34–65.90)	**< 0.001**
***Sex***					
	Men	37.0	36.2	52.0	*NS*
	Women	63.0	63.8	48.0	*NS*
***Area***					
	Urban (%)	49.5	65.2	44.0	**0.010**
	Rural (%)	50.5	34.8	56.0	**0.010**
	Formal educ (%)	62.9	30.4	64.0	**0.010**
	HIV positive (%)	16.6	11.6	4.2	*NS*
***Lifestyle***					
	Current tobacco (%)	51.8	53.6	52.0	*NS*
	Current alcohol (%)	39.2	46.4	36.0	*NS*
	GGT (U/l)	45.15(29.35–85.00)	48.29(31.34–94.51)	73.82(46.00–173.00)	**0.031**
	WPA index	2.87(2.54–3.23)	2.64(2.28–2.90)	2.66(2.42–2.92)	**0.001**
***Body composition***					
	Waist circum. (cm)	77.25(70.20–87.80)	80.18(70.04–87.43)	74.88(64.53–84.74)	*NS*
	Hip circum. (cm)	93.20(84.90–106.1)	93.48(84.75–104.9)	86.85(76.33–98.28)	*NS*
	Waist: hip ratio	0.83 (0.78–0.88)	0.85(0.80–0.90)	0.86 (0.80–0.91)	*NS*
	BMI (kg/m^2^)	22.98(19.31–28.96)	23.53(19.33–28.64)	21.27(16.00–25.51)	*NS*
***CVD measurement***					
	SBP (mmHg)	129 (116–146)	144 (130–171)	127 (106–150)	**<0.001**
	DBP (mmHg)	87 (77–96)	94 (87–108)	80 (72–93)	**<0.001**
	MAP (mmHg)	100 (91–120)	110 (100–132)	96 (87–111)	**<0.001**
	BP medication (%)	7.3	17.4	12.5	**0.002**
	Framingham score	5.0 (3.0–8.0)	10.0 (4.0–16.0)	7.0 (2.5–10.0)	**<0.010**
***Glycaemic status***					
	Glucose (mmol/l)*	4.80 (4.30–5.30)	4.90 (4.00–5.50)	5.00 (4.45–5.35)	*NS*
	HbA1c (%)	5.50 (5.30–5.80)	5.50 (5.20–5.95)	5.40 (5.03–5.98)	*NS*
	Diabetic (%)	5.2	9.7	4.8	**< 0.010**
***Lipid profile***					
	HDL (mmol/l)	1.41 (1.07–1.87)	1.44 (1.00–1.82)	1.61 (1.22–2.25)	*NS*
	LDL (mmol/l)	2.77 (2.07–3.64)	2.91 (2.07–3.51)	2.72 (1.71–3.70)	*NS*
	Trig (mmol/l)	1.07 (0.82–1.54)	1.21 (0.88–1.67)	1.06 (0.80–1.49)	*NS*
	Total chol. (mmol/l)	4.81 (3.40–5.87)	4.98 (4.33–5.98)	4.54 (3.83–6.51)	*NS*
	Trig:HDL	0.78 (0.49–1.25)	0.83 (0.55–1.35)	0.62 (0.46–1.21)	*NS*

Two-tailed hypotheses, z-test and p-values, were calculated between the three groups; only the lowest p-trend was reported between-group differences. Continuous variables reported using median and the range between first quatile and third quartile (Interquartile range Q1-Q3).

**NS**: not significant; **HIV**: Human immunodeficiency virus; **GGT**: gamma-glutamyltransferase; **WPA index**: weighted physical activity index; **HbA1c**: Glycated haemoglobin; **SBP**: systolic blood pressure; **DBP**: diastolic blood pressure; **MAP**: mean arterial pressure; **HDL**: high-density lipoprotein; **LDL**: low-density lipoprotein; **Trig**: triglycerides

* Fasting plasma glucose

Point-biserial correlations were performed to determine the relationship between CV events and modifiable risk factors (Table 2). There was a positive correlation between age and suffering a non-fatal (r_pb_ = 0.126, p < 0.0001) and fatal (r_pb_ = 0.099, p < 0.0001) CV event. Suffering a non-fatal CV event was related to SBP and DBP (r_pb_ = 0.115 and 0.098 with p < 0.0001 for SBP and DBP, respectively). Moreover, WPA and FRS were also significantly associated to non-fatal CVD (r_pb_ = -0.097 and r_pb_ = 0.107 with p < 0.0001 for WPA and FRS, respectively). When looking at fatal CVDs we reported a borderline significant association with WPA (r_pb_ = -0.045, p = 0.050). Among the biochemical risk factors, a positive relationship between fatal CVD and GGT emerged (r_pb_ = 0.067, p = 0.004).

**Table 2:** Point-biserial correlations with 95% confidence limits between CVD outcomes and sample’s features.

	Non-fatal CVD outcomes		Fatal CVD outcomes	
	r_pb_ (95% CI)	Pvalue	r_pb_ (95% CI)	Pvalue
Age	.126 (.083, .169)	**< .0001**	.099 (.056, .143)	**< .0001**
BMI (kg/m^2^)	.004 (-.040, .047)	*NS*	.006 (-.006, .010)	*NS*
WPA-index	-.097 (-.141, -.053)	**< .0001**	-.045 (-.089, .000)	**.050**
FRS	.107 (.064, .150)	**< .0001**	.021 (-.023, .065)	*NS*
SBP (mmHg)	.115 (.072, .158)	**< .0001**	-.010 (-.054, .034)	*NS*
DBP (mmHg)	.098 (.055, .142)	**< .0001**	-.030 (-.074, .014)	*NS*
GGT (U/l)	.014 (-.031, .060)	*NS*	.067 (.022, .112)	**.004**
Fasting glucose (mmol/l)*	.004 (-.042, .050)	*NS*	.009 (-.037, .054)	*NS*
HbA1c (%)	.005 (-.040, .049)	*NS*	-.026 (-.070, .019)	*NS*
Total chol. (mmol/l)	.015 (-.030, .060)	*NS*	-.001 (-.046, .044)	*NS*
Trig (mmol/l)	.040 (-.005, .085)	*NS*	-.003 (-.049, .042)	*NS*

**CVD**: Cardiovascular disease; **BMI**: Body Mass Index; **WPA**: Weighted Physical Activity Index; **FRS**: Framingham Risk Score; **SBP**: Systolic Blood Pression; **DBP**: Dyastolic Blood Pression; **GGT**: Gamma Glutamyltransferase; **HbA1c**: Glycated Haemoglobin; **Total chol.**: Total Cholesterol; **Trig**: Total Tryglicerides; **NS**: Not significant

* Fasting plasma glucose

We observed relevant collinearity among a limited number of potential predictors of cardiovascular disease. In particular, we observed a familiar correlation between systolic blood pressure and diastolic blood pressure. As expected, those variables were inversely correlated to the use of lowering blood pressure drugs. To avoid multicollinearity, a hypertension variable was created according to WHO definition: having systolic blood pressure above 150 mmHg or diastolic blood pressure above 90 mmHg or positive use of lowering blood pressure drugs [43]. Moreover, current smoking and alcohol use also resulted in collinearity, so we merged these two factors to define a four-level variable of dichotomous values defined as current smoking or alcohol use. Several alternative models were performed considering sociodemographics and education, locality, lifestyle and biochemical profiles. According to the LASSO analysis, a comprehensive multivariate model emerged (age, sex, location, education, BMI, Framingham risk score, merged current smoking and alcohol use, hypertension, gamma-glutamyltransferase, fasting glucose, glycated haemoglobin, total cholesterol and total triglycerides). In the second step, comparing three alternative and hierarchical multivariate binomial logistic models, the mutually adjusted, most comprehensive model having all CVD events as outcomes (non-fatal + fatal) was selected over simpler models. In particular, when comparing the simplest vs more complex models, we observed an AUC improvement from 0.732 to 0.776 for the overall CVD outcome. Binary logistic regression analyses were performed (Table 3) to ascertain the effects of modifiable risk factors on the likelihood that participants will suffer from a CV event.

**Table 3:** Odds ratios (OR) and 95% confidence intervals (95% CI) of sample‘s features in relation to non-fatal and fatal cardiovascolar outcomes.

	Non-fatal CVD outcomes		Fatal CVD outcomes	
	OR (95% CI)	Pvalue	OR (95% CI)	Pvalue
Age (5 year increase)	1.87 (1.29, 2.71)	**< .0001**	3.21 (1.86, 5.55)	**< .0001**
Sex (Men vs. Women)	0.68 (0.34, 1.36)	*NS*	1.41 (0.41, 4.85)	*NS*
Location (Urban vs. Rural)	0.92 (0.48, 1.75)	*NS*	1.03 (0.33, 3.23)	*NS*
Education (Any vs. None)	0.55 (0.28, 1.09)	*NS*	1.76 (0.59, 5.31)	*NS*
BMI <18.5 vs. BMI 18.5–25	0.88 (0.38, 2.04)	*NS*	1.65 (0.53, 5.16)	*NS*
BMI 25–30 vs. BMI 18.5–25	0.79 (0.34, 1.80)	*NS*	0.37 (0.04, 3.21)	*NS*
BMI ≥ 30 vs. BMI 18.5–25	0.85 (0.38, 1.90)	*NS*	0.32 (0.03, 3.10)	*NS*
WPA (+ 1 STD)	0.62 (0.46, 0.83)	**0.002**	0.85 (0.50, 1.45)	*NS*
FRS (+ 1 STD)	1.35 (0.90, 2.01)	*NS*	0.49 (0.23, 1.03)	*NS*
S/A-YY vs. S/A-NN	1.62 (0.75, 3.54)	*NS*	0.40 (0.11, 1.49)	*NS*
S/A-YN vs. S/A-NN	1.02 (0.43, 2.41)	*NS*	1.29 (0.28, 5.95)	*NS*
S/A-NY vs. S/A-NN	1.10 (0.37, 3.29)	*NS*	0.70 (0.12, 4.08)	*NS*
Hypertension (Yes vs. No)	2.47 (1.26, 4.85)	**0.009**	0.38 (0.11, 1.27)	*NS*
GGT (+ 1 STD)	0.89 (0.65, 1.21)	*NS*	2.45 (1.36, 4.42)	**0.003**
FGL (+ 1 STD)	0.89 (0.68, 1.17)	*NS*	1.25 (0.70, 2.24)	*NS*
GHB (+ 1 STD)	0.82 (0.62, 1.09)	*NS*	0.60 (0.34, 1.04)	*NS*
TCH (+ 1 STD)	0.75 (0.55, 1.03)	*NS*	1.01 (0.56, 1.82)	*NS*
TGL (+ 1 STD)	1.10 (0.79, 1.53)	*NS*	0.96 (0.50, 1.83)	*NS*

**CVD**: Cardiovascular disease; **OR**: Mutually adjusted Odds Ratio; **BMI**: Body Mass Index; **WPA**: Weighted Physical Activity Index; **FRS**: Framingham Risk Score; **S/A**: Smoking and alcohol; **Y**: Yes; **N**: No; **GGT**: Gamma Glutamyl Transferase; **FGL**: Fasting Glucose; **GHB**: Glycated Haemoglobin; **TCH**: Total Cholesterol; **TGL**: Total tryglicerides.

According to our analyses, a 5-year increase in age would result in an increased odd of CVD, ranging between 87% for non-fatal CVD to more than threefold for fatal CVD. On the contrary, among other demographics and general features, sex, education and location were not related with a significant odd for CVD. More than twofold higher odd of non-fatal CVD was also reported for subjects with hypertension (comprehensive of subjects taking blood pressure-lowering drugs) versus subjects without hypertension. When considering the biochemical profile, one standard deviation increase of gamma-glutamyltransferase was related to two and half time increased odd of fatal CVD outcome. Finally, multiplicative interaction terms between the above-reported variables did not result as statistically significant.

**Fig 1:**
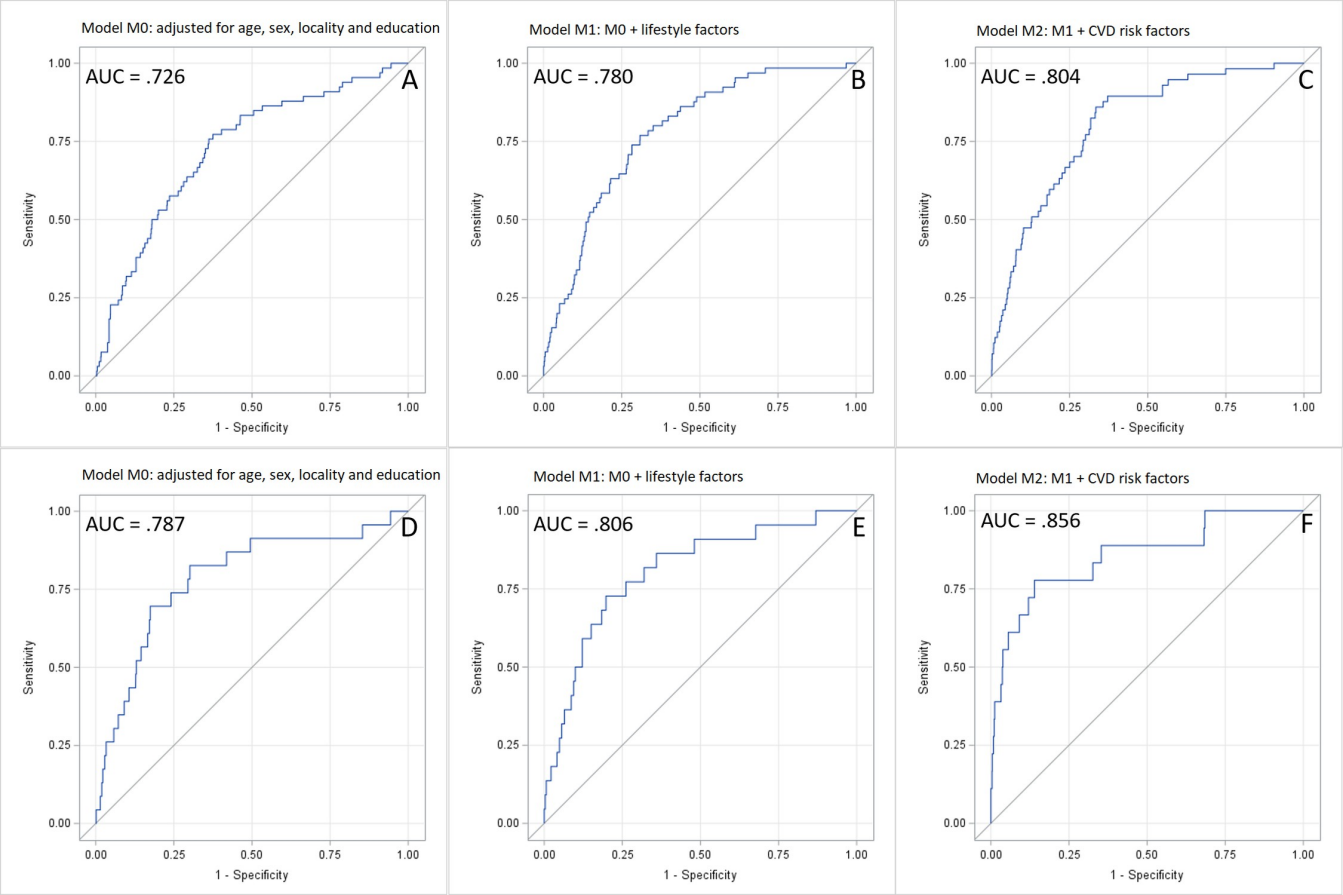
ROC curve of risk factors related to all, non-fatal and fatal CV outcomes. M0 (panels A and D): model of age, sex, locality and education; M1 (panels B and E): M0 supplementary adjusted for lifestyle (merged current smoking and alcohol use and weighted physical activity index); M2 (panels C and F): M1 supplementary adjusted for CVD risk factors (Framingham risk score and hypertension) and biochemical profile.

**Fig 2:**
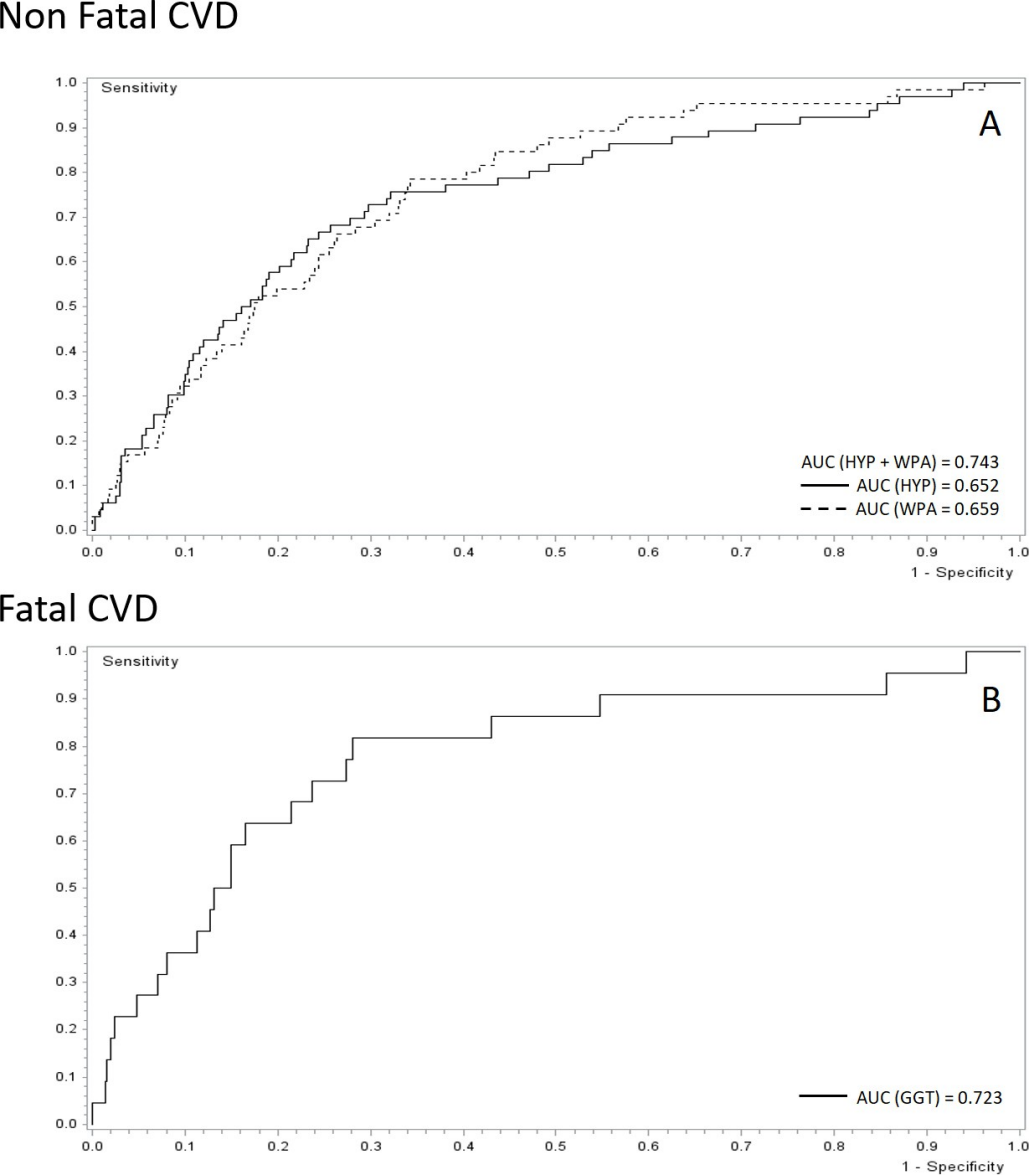
ROC curve of risk factors related to non-fatal and fatal CV outcomes. HYP: Hypertension, WPA: Weighted Physical Activity Index, GGT: Gamma-glutamyl-transferase.

When looking at the results from ROC curve analysis, we observe that sequential modelling with the inclusion of behavioural and biochemical profile variables improves the model (Fig 1). Specifically, when considering non-fatal CVD events, we observed that the inclusion of behavioural (Fig 1-Panel B) and biochemical profile variables (Fig 1-Panel C) resulted in an AUC of 0.780 and 0.804, respectively. Furthermore, a more considerable increase concerning the AUC = 0.726 of a simple model based on socio-demographic factors (Fig 1-Panel A). Similarly, when looking at models of fatal CVD, we observed an improvement of the AUC from 0.787, resulting in the simplest model based on socio-demographic factors (Fig 1-Panel D) to 0.806 to 0.856 when considering models further considering behavioural (Fig 1-Panel E) and biochemical profile variables (Fig 1-Panel F), respectively. Finally, a limited number of selected modifiable risk factors are capable of predicting CVD outcomes. Specifically, a satisfactory association with non-fatal CVD was observed for hypertension (AUC = 0.652) and physical activity (AUC = 0.659), the comprehensive model having both predictors had an AUC of 0.743 (Fig 2, panel A). When looking at fatal CVD, the only predictor was GGT with an AUC = 0.723 (Fig 2, panel B).

## Discussion

The central aim of this research was to identify the 5-year prognostic value of modifiable risk factors for non-fatal and fatal CV events within a selected group of South Africans. Our analyses suggest that hypertension, GGT and weighted physical activity are associated with cardiovascular outcomes. On the one hand, we confirmed how hypertension and physical activity are related to non-fatal CVD outcomes when explicitly looking at the type of CVD outcome. On the other hand, it seems that only gamma-glutamyltransferase was a predictor of fatal events. The existing CV management tools (PC101, EDL, ICDM) used within the South African public health system present a risk assessment of modifiable and non-modifiable risk factors (presented as an integrated process) for especially myocardial infarction over ten years, and do not differentiate between risks for fatal or non-fatal CV events. This is of great importance since the aetiology of fatal and non-fatal CV outcomes may be due to entirely different aetiology [44].

Our results confirm the significant role of age in developing CV events. Participants who suffered fatal CV events were older (median age of 60.43 years) than those who suffered non-fatal CV events (average age of 57.12 years). Interestingly, the prevalence of HIV positive participants was higher in those with no cardiovascular events. This finding is in contrast with previous findings by Hyle et al. [45], who found that due to improvements in curbing communicable diseases like HIV and freely available antiretroviral treatment [46], people are becoming more prone to developing CVDs because they are living longer [47]. An increased life expectancy has been proven to increase the risk of developing CVDs due to physical inactivity and a weakened immune system in older people [48]. However, CVDs do not only affect older people [49]. Most people suffer from CVDs in their youth because of poor lifestyle choices and proceed with these CVDs into adulthood [49]. Because the immune system is still robust at an early age, a CV episode at a younger age may, therefore, result in a non-fatal event [48, 50]. Interestingly, results from a study conducted in Uganda (East-Central Africa) revealed that people living with HIV have 30% lower odds of having hypertension [51]. This observation of Okello et al. [51] is aligned with a meta-analysis [52] of studies conducted in sub-Saharan Africa examining the association between HIV and cardiometabolic traits. The results from the meta-analysis found that HIV infection is associated with lower SBP as well as DBP. Given the fact that hypertension emerged as a significant risk factor for non-fatal CV outcomes, a possible explanation for the low prevalence of HIV infected participants observed in this CV outcome group might be that HIV infected participants within our study population did not present with elevated blood pressure levels. However, more comprehensive analyses of the data are needed to clarify this observation, but that is beyond the scope of this study.

Rural areas reported a higher prevalence of fatal CV events, whereas urban areas reported higher non-fatal CV events. These results agree with findings from Kapral et al. [53], who reported that CV-related mortalities were higher in rural than in urban communities. Other studies also reported that, although CV-related mortalities were higher in rural than in urban communities, the risk-factor burden was higher in urban than in rural communities in the countries included in their studies [53, 54]. Although locality was not a significant predictor for CV events, a higher prevalence of fatal CV events was observed in the rural community than in urban areas. The reason for this *status quo* could be the difficulty in accessing preventative health care programmes and that health care services generally remain substantially underdeveloped [55]. Also, rural populations are generally more resource-constrained than their urban counterparts, making them more vulnerable to social determinants of health and less likely to have the means to access care [56]. Some barriers faced by people in the rural communities of South Africa, and which could affect the high CV mortality rates reported in these areas, have been listed. These barriers include (i) cost and time for patients travelling long distances to access services are more significant for rural people, (ii) higher cost and time of conducting outreach services and the resulting need for more healthcare workers per capita compared to urban areas, (iii) diseconomies of scale exist, making the cost of delivering services per capita higher, (iv) ambulances take longer to reach patients, (v) healthcare workers may be reluctant to live in rural areas as these are often far from desirable amenities (schools, banks, malls, gyms), and (vi) fewer opportunities exist for the employment of family members, e.g. a spouse [57]. The role of COPC and WBOTs in cardiovascular health promotion of patients with modifiable and non-modifiable risk factors for fatal and non-fatal CV events necessitates attention.

This current study also found a relationship between CV events and having some form of education. It can be assumed that people who attained formal education are more likely to reside in urban areas where the socio-economic status is higher compared to those in rural areas [58]. Individuals with some education level have relatively greater knowledge about disease conditions than those without formal education [57]. Education plays a role in socio-economic status; people who attained higher education have better occupational and economic opportunities than those who obtained lower/no education levels, impacting their quality of life [58]. Health literacy and knowledge of cardiovascular risk factors may encourage people to make behavioural changes and reduce risk factors within the communities [57, 58]. Health literacy and education levels were not explicitly listed within the existing South African CVD management tools (Primary Care 101(PC101) [59] and the Essential Drug List (EDL)) [60]. However, the Integrated Chronic Disease Management model of care [61] does reiterate the importance of empowering patients with chronic diseases towards assisted self-management.

Elevated blood pressure is an essential modifiable CV risk factor, especially in black populations [62]. The South African National Health and Nutrition Survey (SANHANES) reported an increased prevalence of hypertension, especially in the elderly [63]. Our results from ROC curve analyses identified hypertension as an essential predictor for non-fatal CV events. These findings add to the longstanding knowledge that the control of high blood pressure and its risk factors deserve attention in the black population [64].

Evidence exists in support of both SBP and DBP as essential measures of CV risk but at different ages of an individual [65, 66]. DBP increases until approximately the age of 50, whereafter it tends to level off over the next decade and may remain the same or decrease further later in life, whereas SBP continues to increase throughout life [65]. Therefore, DBP seems to be a more potent CV risk factor than SBP until the age of 50 years, and after that, SBP becomes more critical [64]. This is particularly relevant to sub-Saharan Africa, particularly in South Africa, where life expectancy has improved beyond the age of 50 [67]. In addition, controlling SBP reduces total mortality, cardiovascular mortality, stroke and heart failure events [65].

Our findings of the association of CV events with the use of antihypertensive medication may suggest that factors other than traditional risk factors may be necessary for determining outcomes in this area of study. Possible contributing factors could include access to and affordability of health services and medications, thresholds for diagnoses and treatments, and the population’s educational level [63]. There may be more significant differences between urban and rural communities, including their level of education, and access to, quality of, and affordability of health care, which may contribute to higher rates of death from CVDs in rural areas, despite a lower risk factor burden [49, 55, 62]. The social determinants of health remain a taxonomy to comprehend the multifaceted realities in managing CVDs, especially in cardiovascular health promotion.

## Study limitations and strengths

Considering that data were collected at different intervals, there may have been concerns with missing data. Some variables did not show a normal distribution, but the use of rank transformation allowed us to manage such an issue. In addition, the sampling framework was not nationally representative, and therefore, the information from this study cannot be used to represent the entire country’s status. Finally, the limited number of fatal CVD negatively influenced the power to detect associations so that a possible number of false-negative results arose. However, this study’s findings impact the community and primary healthcare practice, research, education, and policy development, contributing to primary prevention and control of the prevalence of CVDs in South Africa. It is also recommended that the ICDM model of South Africa ensures that CVD prevention is included in HIV prevention through patient-centred health education. Furthermore, health promoters should be informed about these results to enrich the health promotion awareness campaigns on the primary health care level.

## Conclusion

We found that high blood pressure, GGT, and physical activity have significant prognostic values for fatal and non-fatal cardiovascular events in the research population. These findings further emphasise the importance of highlighting health behaviours (modifiable risk factors) when planning this population’s cardiovascular health education and intervention programmes. The South African health system’s response to the rising CVD burden is reflected in various care initiatives such as PC101, ICDM model of care, and risk assessment within the EDL. The current care initiatives integrate risk factors, whether modifiable or non-modifiable and acknowledge the role of assisted self-management of CVDs. However, considering the complexity of CVD integrated into the social determinants of health, patients with CVD are still falling through the cracks in the current health system. Therefore, more focused cardiovascular health promotion is recommended to (i) contextualise physical activity index to adapt to the robust South African health context, (ii) add alcohol abuse and sedentary lifestyle to the major risk factors for CVDs in the EDL, (iii) reduce the time frames for risk assessment for myocardial infarction and CVD risks from 10 years to 5 years, (iv) strengthen cardiovascular health promotion interventions through Community Orientated Primary Care, and Ward Based Outreach Teams on an individual level through motivational interviewing rather than one-directional health education, (v) establish cardiovascular health promotion interventions aimed to enhance not only assisted self-management, as highlighted within the ICDM model of care but to enhance cardiovascular self-care and (vi) highlight the trilogy of hypertension, physical inactivity and alcohol consumption in cardiovascular health promotion.

Thus, to conclude, it is essential to fortify established risk assessment and CVD management tools, currently used in South African health promotion strategies, with the trilogy of hypertension, physical inactivity and alcohol consumption to enhance self-care of CVDs and build on the established health system.

## Supporting information

S1 File PURE adults questionnaire. (PDF)

S2 File PURE physical activity questionnaire. (PDF)

S1 Dataset (CSV)

## References

[1] Hamid S, Groot W, Pavlova M. Trends in cardiovascular diseases and associated risks in sub-Saharan Africa: a review of the evidence for Ghana, Nigeria, South Africa, Sudan and Tanzania. Aging Male. 2019 Sep;22(3):169–76. doi: 10.1080/13685538.2019.1582621 30879380

[2] Bosire EN, Cohen E, Erzse A, Goldstein SJ, Hofman KJ, Norris SA. ’I’d say I’m fat, I’m not obese’: obesity normalisation in urban-poor South Africa. Public Health Nutr. 2020 Jun;23(9):1515–26. doi: 10.1017/S1368980019004440 32200768 PMC10200575

[3] Gouda HN, Charlson F, Sorsdahl K, Ahmadzada S, Ferrari SA, Erskine H, et al. Burden of non-communicable diseases in sub-Saharan Africa, 1990–2017: results from the Global Burden of Disease Study 2017. Lancet Glob Health. 2019 Oct;7(10):e1375–87. doi: 10.1016/S2214-109X(19)30374-2 31537368

[4] Yuyun MF, Sliwa K, Kengne AP, Mocumbi AO, Bukhman G. Cardiovascular diseases in Sub-Saharan Africa compared to high-income countries: an epidemiological perspective. Glob Heart. 2020 Feb;15(1): Article 15 [18 p.]. doi: 10.5334/gh.403 32489788 PMC7218780

[5] Basu S, Wagner RG, Sewpaul R, Reddy P, Davies J. Implications of scaling up cardiovascular disease treatment in South Africa: a microsimulation and cost-effectiveness analysis. Lancet Glob Health. 2019 Feb;7(2), 270–80. doi: 10.1016/S2214-109X(18)30450-9 30528531

[6] Kabudula CW, Houle B, Collinson MA, Kahn K, Gómez-Olivé FX, Clark SJ, et al. Progression of the epidemiological transition in a rural South African setting: findings from population surveillance in Agincourt, 1993–2013. BMC Public Health, 2017 May;17: Article 424 [15 p.]. doi: 10.1186/s12889-017-4312-x 28486934 PMC5424387

[7] Kwan GF, Mayosi BM, Mocumbi AO, Miranda JJ, Ezzati M, Jain Y, et al. Endemic cardiovascular diseases of the poorest billion. Circulation, 2016 Jun;133(24): 2561–75. doi: 10.1161/CIRCULATIONAHA.116.008731 27297348

[8] Omodan BI, Dube B, Tsotetsi CT. Decolonising the rural-urban dichotomy in South Africa on asset-based approach-research. Progressio, 2019 Jan;41:article 5665 [17 p.]. doi: 10.25159/0256-8853/5665

[9] Said-Mohamed R, Prioreschi A, Nyati LH, Munthali RJ, Kahn K, Tollman SM, et al. Rural-urban variations in age at menarche, adult height, leg-length and abdominal adiposity in black South African women in transitioning South Africa. Ann Hum Biol. 2018 Mar;45(2):123–32. doi: 10.1080/03014460.2018.1442497 29557678 PMC5964443

[10] Statistics South Africa. Mortality and causes of death in South Africa: Findings from death notification. Statistical release (P0309.3). [Internet]. 2017 [cited 2021 Aug 21]. Available from: https://www.statssa.gov.za/publications/P03093/P030932017.pdf

[11] Keates AK, Mocumbi AO, Ntsekhe M. Cardiovascular disease in Africa: epidemiological profile and challenges. Nat Rev Cardiol. 2017 May;14(5):273–93. doi: 10.1038/nrcardio.2017.19 28230175

[12] Woudberg NJ, Goedecke JH, Lecour S. Protection from cardiovascular disease due to increased high-density lipoprotein cholesterol in African black populations: myth or reality? Ethn Dis. 2016 Oct 20;26(4):553–60. doi: 10.18865/ed.26.4.553 27773983 PMC5072485

[13] Statistics South Africa. General household survey. Statistical release (P0318). [Internet]. 2018 [cited 2021 Feb 18]. Available from: http://www.statssa.gov.za/publications/P0318/P03182018.pdf

[14] Dorrington RE, Bradshaw D, Laubscher R, Nannan N. Rapid mortality surveillance report 2017. [Internet]. Cape Town: South African Medical Research Council. 2019 [cited 2021 Feb 8]. Available from: https://www.samrc.ac.za/sites/default/files/files/2019-02-06/RapidMortalitySurveillanceReport2017.pdf

[15] Micklesfield LK, Kagura J, Munthali R, Crowther NJ, Jaff N, Gradidge P, et al. Demographic, socio-economic and behavioural correlates of BMI in middle-aged black men and women from urban Johannesburg, South Africa. Glob Health Action. 2018 Aug;11(sup2): article 1448250 [13 p.]. doi: 10.1080/16549716.2018.1448250 30079826 PMC6084500

[16] Mosadeghrad MA, Gebru AA, Sari AA, Tafesse TB. Impact of food insecurity and malnutrition on the burden of non-communicable diseases and death in Ethiopia: a situational analysis. Hum Antibodies. 2019 Nov;27(4):213–20. doi: 10.3233/HAB-190369 30958340

[17] Burger A, Pretorius R, Fourie CMT, Schutte AE. The relationship between cardiovascular risk factors and knowledge of cardiovascular disease in African men in the North-West Province. Health SA Gesondheid, 2016 Oct;21:364–71. doi: 10.1016/j.hsag.2016.07.003

[18] Gbadamosi MA, Tlou B. Modifiable risk factors associated with non-communicable diseases among adult outpatients in Manzini, Swaziland: a crosssectional study. BMC Public Health. 2020 May 12;20(1):665. doi: 10.1186/s12889-020-08816-0 32398061 PMC7216325

[19] World Health Organization. Non-communicable Diseases (NCD) Country Profiles. [Internet]. 2018 [cited 2021 Feb 18]. Available from: https://www.who.int/nmh/countries/zaf_en.pdf?ua=1

[20] Hauser A, Kusejko K, Johnson LF, Wandeler G, Riou J, Goldstein F, et al. Bridging the gap between HIV epidemiology and antiretroviral resistance evolution: modelling the spread of resistance in South Africa. PLoS Comput Biol. 2019 Jun 24;15(6):e1007083 [17 p.]. doi: 10.1371/journal.pcbi.1007083 31233494 PMC6611642

[21] Department of Health. The national health promotion policy and strategy. [Internet]. 2015 [cited 2021 Feb 8]. Available from: https://health-e.org.za/wp-content/uploads/2015/09/The-National-Health-Promotion-Policy-and-Strategy.pdf

[22] Teo K, Chow CK, Vaz M, Rangarajan S, Yusuf S. The Prospective Urban Rural Epidemiology (PURE) study: examining the impact of societal influences on chronic non-communicable diseases in low-, middle-, and high-income countries. Am Heart J. 2009 Jul;158(1):1-7.e1. doi: 10.1016/j.ahj.2009.04.019 19540385

[23] Breet Y, Lackland DT, Ovbiagele B, Owolabi MO, Ogedegbe G, Kruger IM, et al. Is the cardiovascular health of South Africans today comparable with African Americans 45 years ago? J Hypertens. 2019 Aug;37(8):1606–14. doi: 10.1097/HJH.0000000000002082 30950976

[24] Breet Y, Schutte AE, Huisman HW, Eloff FC, Du Plessis JL, Kruger A, et al. Lung function, inflammation and cardiovascular mortality in Africans. Eur J Clin Invest. 2016 Nov;46(11):901–10. doi: 10.1111/eci.12674 27600376

[25] Maritz M, Fourie CMT, Van Rooyen JM, Schutte AE. Evaluating several biomarkers as predictors of aortic stiffness in young and older Africans, not consuming alcohol based on self-report. Diabetes Res Clin Pract. 2018 Aug;142:312–20. doi: 10.1016/j.diabres.2018.05.048 29906479

[26] Nienaber-Rousseau C, Sotunde OF, Ukegbu PO, Myburgh PH, Wright HH, Havemann-Nel L, et al. Socio-demographic and lifestyle factors predict 5-year changes in adiposity among a group of black South African adults. Int. J. Environ. Res. Public Health 2017 Sep;14(9), 1089 [16 p.]. doi: 10.3390/ijerph14091089 28930196 PMC5615626

[27] Ojwang AA, Kruger HS, Zec M, Ricci C, Pieters M, Kruger IM, et al. Plasma phospholipid fatty acid patterns are associated with adiposity and the metabolic syndrome in black South Africans: a cross-sectional study. Cardiovasc J Afr. 2019 Jul/Aug 23;30(4):228–38. doi: 10.5830/CVJA-2019-026 31361296

[28] Schutte AE, Conti E, Mels CMC, Smith W, Kruger R, Botha S, et al. Attenuated IGF-1 predicts all-cause and cardiovascular mortality in a Black population: A five-year prospective study. Eur J Prev Cardiol. 2016 Nov;23(16):1690–99. doi: 10.1177/2047487316661436 27450159

[29] Zatu MC, Van Rooyen JM, Kruger A, Schutte AE. Alcohol intake, hypertension development and mortality in black South Africans. Eur J Prev Cardiol. 2016 Feb;23(3):308–15. doi: 10.1177/2047487314563447 25500903

[30] Pisa PT, Behanan R, Vorster HH, Kruger A. Social drift of cardiovascular disease risk factors in Africans from the North West Province of South Africa: the PURE study. Cardiovasc J Afr. 2012 Aug;23(7):371–8. doi: 10.5830/CVJA-2012-018 22914994 PMC3721859

[31] Von Elm E, Altman DG, Egger M, Pocock SJ, Gøtzsche PC, Vandenbroucke JP. The strengthening the reporting of observational studies in epidemiology (STROBE) statement: guidelines for reporting observational studies. J Clin Epidemiol. 2008 Apr;61(4):344–9. doi: 10.1016/j.jclinepi.2007.11.008 18313558

[32] Simera I, Moher D, Hoey J, Schulz K, Altman D. A catalogue of reporting guidelines for health research. Eur J Clin Invest. 2010 Jan;40(1):35–53. doi: 10.1111/j.1365-2362.2009.02234.x 20055895

[33] Baecke JA, Burema J, Frijters JE. (1982) A short questionnaire for the measurement of habitual physical activity in epidemiological studies. Am. J. Clin. Nutr. 1982 Nov;36(5):936–42. doi: 10.1093/ajcn/36.5.936 7137077

[34] Kruger H.; Venter C.; Steyn H.S. A standardised physical activity questionnaire for a population in transition:The THUSA study. Afr. J. Phys. Health Educ. Recreat. Dance 2000;6:54–64.

[35] Stewart A, Marfell-Jones M, International Society for Advancement of Kinanthropometry. International standards for anthropometric assessment. Lower Hutt: International Society for the Advancement of Kinanthropometry; 2011.

[36] World Health Organization. Obesity: preventing and managing the global epidemic. [Internet]. Geneva: World Health Organization. 2000 [cited 2021 Feb 18]. Available from: https://www.who.int/nutrition/publications/obesity/WHO_TRS_894/en/11234459

[37] Friedewald WT, Levy RI, Fredrickson DS. Estimation of the concentration of low-density lipoprotein cholesterol in plasma, without use of the preparative ultracentrifuge. Clin Chem. 1972 Jun;18(6):499–502. 4337382

[38] Jahangiry L, Farhangi MA, Rezaei F. Framingham risk score for estimation of 10-years of cardiovascular diseases risk in patients with metabolic syndrome. J Health Popul Nutr. 2017 Nov 13;36(1): article 36 [6 p.]. doi: 10.1186/s41043-017-0114-0 29132438 PMC5682637

[39] Lee MS, Flammer AJ, Li J, Lennon RJ, Delacroix S, Kim H, et al. Comparison of time trends of cardiovascular disease risk factors and Framingham risk score between patients with and without acute coronary syndrome undergoing percutaneous intervention over the last 17 Years: from the Mayo clinic percutaneous coronary intervention registry. Clin Cardiol. 2015 Dec;38(12): 747–56. doi: 10.1002/clc.22484 26671071 PMC6490742

[40] Anderson TJ, Gregoire J, Pearson GJ. Canadian cardiovascular society guidelines for the management of dyslipidemia for the prevention of cardiovascular disease in the adult. Can J Cardiol. 2016 Nov;32(11):1263–82. doi: 10.1016/j.cjca.2016.07.510 27712954

[41] Gibbs BB, King WC, Belle SH, Jakicic JM. Six-month changes in ideal cardiovascular health vs. Framingham 10-year coronary heart disease risk among young adults enrolled in a weight loss intervention. Prev Med. 2016 May;86:123–9. doi: 10.1016/j.ypmed.2016.02.033 26923555 PMC4837008

[42] Blom G. Statistical estimates and transformed beta-variables. [Internet] [PhD dissertation]. [Stockholm]; 1958 [cited 2021 Aug 8]. Available from: http://urn.kb.se/resolve?urn=urn:nbn:se:su:diva-75457

[43] Moser M. World Health Organization-International Society of Hypertension Guidelines for the Management of Hypertension-Do These Differ From the U.S. Recommendations? Which Guidelines Should the Practicing Physician Follow?. J Clin Hypertens (Greenwich). 1999 Jul;1(1):48–54. 11416593

[44] Ricci C, Wood A, Muller D, Gunter M J, Agudo A, Boeing H et al. Alcohol intake in relation to non-fatal and fatal coronary heart disease and stroke: EPIC-CVD case-cohort study. BMJ. 2018 May 29;361:k934 [9 p.]. doi: 10.1136/bmj.k934 29844013 PMC5972779

[45] Hyle EP, Mayosi BM, Middelkoop K, Mosepele M, Martey MB, Walensky RP, et al. The association between HIV and atherosclerotic cardiovascular disease in sub-Saharan Africa: a systematic review. BMC Public Health. 2017 Dec 15;17(1): article 954 [15 p.]. doi: 10.1186/s12889-017-4940-1 29246206 PMC5732372

[46] Fabian J, Maher HA, Clark C, Naicker S, Becker P, Venter WDF. Morbidity and mortality of black HIV-positive patients with end-stage kidney disease receiving chronic haemodialysis in South Africa. S Afr Med J. 2015 Jan;105(2):110–14. doi: 10.7196/samj.8369 26242528

[47] Feinstein MJ, Hsue PY, Benjamin LA, Bloomfield GS, Currier JS, Freiberg MS, et al. Characteristics, prevention, and management of cardiovascular disease in people living with HIV a scientific statement from the American heart association. Circulation, 2019 Jun;140(2): 98–124. doi: 10.1161/CIR.0000000000000695 31154814 PMC7993364

[48] Anderson E, Durstine JL. Physical activity, exercise, and chronic diseases: a brief review. Sports Med Health Sci, 2019 Dec;1(1):3–10. doi: 10.1016/j.smhs.2019.08.006 35782456 PMC9219321

[49] Chen C, Jin Y, Lo IL, Zhao H, Sun B, Zhao Q, et al. Complexity change in cardiovascular disease. Int J Biol Sci. 2017 Oct 17;13(10):1320–8. doi: 10.7150/ijbs.19462 29104498 PMC5666530

[50] Latif Z, Garg N. The impact of marijuana on the cardiovascular system: a review of the most common cardiovascular events associated with marijuana use. J. Clin. Med. 2020 June;9(6):e1925 [16 p.]. doi: 10.3390/jcm9061925 32575540 PMC7355963

[51] Okello S, Ueda P, Kanyesigye M, Byaruhanga E, Kiyimba A, Amanyire G, et al. (2017). Association between HIV and blood pressure in adults and role of body weight as a mediator: cross-sectional study in Uganda. J Clin Hypertens (Greenwich). 2017 Nov;19(11):1181–91. doi: 10.1111/jch.13092 28895288 PMC5693777

[52] Dillon DG, Gurdasani D, Riha J, Ekoru K, Asiki G, Mayanja BN, et al. Association of HIV and ART with cardiometabolic traits in sub-Saharan Africa: a systematic review and meta-analysis. Int J Epidemiol. 2013 Dec;42(6):1754–71. doi: 10.1093/ije/dyt198 24415610 PMC3887568

[53] Kapral MK, Austin PC, Jeyakumar G, Hall R, Chu A, Khan AM, et al. Rural-urban differences in stroke risk factors, incidence, and mortality in people with and without prior stroke the CANHEART stroke study. Circulation. 2019 Feb;12(2), e004973 [10 p.]. doi: 10.1161/CIRCOUTCOMES.118.004973 30760007

[54] Gupta V, Millett C, Walia GK, Kinra S, Aggarwal A, Prabhakaran P, et al. Socio-economic patterning of cardiometabolic risk factors in rural and peri-urban India: Andhra Pradesh children and parents study (APCAPS). J Public Health. 2015 Mar;23(3):129–36. doi: 10.1007/s10389-015-0662-y 26000232 PMC4434856

[55] Vergunst R, Swartz L, Mji G, MacLachlan M, Mannan H. ’You must carry your wheelchair’ barriers to accessing healthcare in a South African rural area. Glob Health Action. 2015 Oct 1;8: article 29003 [9 p.]. doi: 10.3402/gha.v8.29003 26434691 PMC4592846

[56] Gyasi RM, Phillips DR. Demography, socio-economic status and health services utilisation among older Ghanaians: implications for health policy. Ageing Int. 2020 Mar;45(1):50–71. doi: 10.1007/s12126-018-9343-9

[57] Kapwata T, Manda S. Geographic assessment of access to health care in patients with cardiovascular disease in South Africa. BMC Health Serv Res. 2018 Mar;18: article 197 [10 p.]. doi: 10.1186/s12913-018-3006-0 29566692 PMC5863828

[58] Vellakkal S, Millett C, Basu S, Khan Z, Aitsi-Selmi A, Stuckler D, et al. Are estimates of socio-economic inequalities in chronic disease artefactually narrowed by self-reported measures of prevalence in low-income and middle-income countries? Findings from the WHO-SAGE survey. J Epidemiol Community Health. 2015 Mar;69(3):218–25. doi: 10.1136/jech-2014-204621 25550454 PMC4345525

[59] Department of Health. Primary care 101: Guideline 2013/14. [Internet]. 2013 [cited 2021 Feb 8]. Available from: https://health-e.org.za/wp-content/uploads/2015/04/PC-101-Guideline-v2-2013-14-2.pdf

[60] Department of Health. Standard treatment guidelines and essential medicines list for South Africa. [Internet]. 2019 [cited 2021 Aug 21]. Available from: https://www.sapc.za.org/Media/Default/Documents/STG%20hospital%20level%20adult%202019_v2.0.pdf

[61] Department of Health. Integrated chronic diseases management manual. [Internet]. 2015 [cited 2021 Feb 8]. Available from: http://www.kznhealth.gov.za/family/Integrated-chronic-disease-management-manual.pdf

[62] Xhakaza L, Abrahams-October Z, Mohammednur MM, Pearce B, Adeniyi OV, Johnson R, et al. Socio-demographic and modifiable risk factors of diabetes and hypertension among resource constrained patients from rural areas in Mdantsane Township in South Africa. Afr Health Sci. 2020 Sep;20(3):1344–54. doi: 10.4314/ahs.v20i3.41 33402984 PMC7751544

[63] Berry KM, Parker W, Mchiza ZJ, Sewpaul R, Labadarios D, Rosen S, et al. Quantifying unmet need for hypertension care in South Africa through a care cascade: evidence from the SANHANES, 2011–2012. BMJ Global Health, 2017 Aug;2(3): e000348 [10 p.]. doi: 10.1136/bmjgh-2017-000348 29082013 PMC5656122

[64] Zheng J, Sun Z, Guo X, Xie Y, Sun Y, Zheng L. Blood pressure predictors of stroke in rural Chinese dwellers with hypertension: a large-scale prospective cohort study. BMC Cardiovasc Disord, 2019 Aug;19:article 206 [7 p.]. doi: 10.1186/s12872-019-1186-0 31464591 PMC6716914

[65] Beddhu S, Chertowm GM, Greene T, Whelton PK, Ambrosius WT, Cheung AK, et al. Effects of intensive systolic blood pressure lowering on cardiovascular events and mortality in patients with type 2 diabetes mellitus on standard glycemic control and in those without diabetes mellitus: Reconciling results from ACCORD BP and SPRINT. J Am Heart Assoc. 2018 Sep 18;7(18):e009326. doi: 10.1161/JAHA.118.009326 30371182 PMC6222943

[66] Flint AC, Conell C, Renm X. Effect of systolic and diastolic blood pressure on cardiovascular outcomes. N Engl J Med. 2019 Jul;381(3):243–51. doi: 10.1056/NEJMoa1803180 31314968

[67] Chirinda W, Phaswana-Mafuya N. Happy life expectancy and correlates of happiness among older adults in South Africa. Aging Ment Health. 2019 Aug;23(8):1000–7. doi: 10.1080/13607863.2018.1471581 29781714

