# Supplementary material for: Prognostic values of modifiable risk factors for cardiovascular events in South African health promotion
Source: PLoS One. 2022 Aug 10;17(8):e0271169. doi: 10.1371/journal.pone.0271169 (PMC9365156; doi:10.1371/journal.pone.0271169)
Supplement: S1 File — (PDF) [file pone.0271169.s001.pdf]

PURE/South Africa

We are very grateful to you for your participation in this study. All information given by you will be held in strict confidence, and will be used for the purpose of this study only after removing any personal identifying information.

Adult Questionnaire

INSTRUCTIONS

Please answer EACH question by marking an X in ONE BOX on each line:
(unless otherwise instructed)

[X]

OR

By writing number(s) in the spaces provided:

[1] [8]

OR

By specifying the answer on the line(s) provided

April 28, 2005

Adult Questionnaire

Subject Initials- F= first letter of first name
M= first letter of middle name
L= first letter of last name

3. National I.D#

If not applicable please mark the N/A box

Ethnicity Codes

01 - South Asian (India, Sri Lanka, Pakistan, Bangladesh)

02 - Chinese (China, Hong Kong, Taiwan)

03 - Japanese

04 - Malays

05 - Other Asian (Korea, Malaysia, Papua New Guinea, Thailand, Philippines, Indonesia, Nepal,Vietnam, Cambodia, Laos, Myanmar/Burma, Bhutan, Singapore)

06 - Persian

07 - Arab

08 - Black African

09 - Coloured African (Subsaharan African only)

10 - European

11 - Native North/South American or Australian Aborigine

12 - Latin American (Latino)

13 - Bantu/Semi Bantu

14 - Hemitic/Semi Hemitic

15 - Nilotic/Hausa

16 - Pygmie

17 - Swahili

18 - Other (any other ethnoracial group not listed above)

Subject ID

☐☐ *Centre #* ☐☐☐ *Community#* ☐☐☐ *Household #* ☐☐ *Subject #*

Subject Initials ☐☐☐ *F M L*

Today's date: ☐☐☐☐ *year* ☐☐ *month* ☐☐ *day*

1. **Name:**____________________ *Given name* ____________________ *Surname*

2. **Not applicable in South Africa**

3. **National identity # or equivalent:** ______________________________ **N/A** ☐

4. **DOB:** ☐☐☐☐ *year* ☐☐ *month* ☐☐ *day* **OR** **Age** ☐☐ yrs

5. **Sex:** ☐ Female ☐ Male

6. **Marital status:** (check one only)

☐ Never married ☐ Currently married ☐ Common law/Living with partner
☐ Widowed ☐ Separated ☐ Divorced

7. **Ethnicity:** ☐☐ ⟶ (Please refer to facing page for codes)

8. **Caste/Tribe:** ____________________

9. **What level of formal education have you <u>completed</u>?** (check highest level only):

☐ None
☐ Primary
☐ Secondary/highschool/higher secondary
☐ Trade School
☐ College/University
☐ Unknown

Adult Questionnaire

11. Occupation

Group 1: Legislators, senior officials and managers

Legislators and senior officials
Corporate managers
General managers
Businessman

Group 2: Professionals

Physical, mathematical and engineering science professionals
Life science and health professionals
Teaching professionals
Other professionals

Group 3: Technicians and associate professionals

Physical, mathematical and engineering-science associate professionals/technicians
Life science and health associate professionals/technicians
Teaching associate professionals/technicians
Other associate professionals/technicians

Group 4: Clerks

Clerks
Customer service clerks

Group 5: Service workers and shop and market sales workers

Personal and protective services workers
Models, salespersons and demonstrators

Group 6: Skilled agricultural and fishery workers

Market-oriented skilled agricultural and fishery workers
Subsistence agricultural and fishery workers

Group 7: Craft and related trade workers

Extraction and building trade workers
Metal, machinery and related trades workers
Precision, handicraft, printing and related trades workers
Other craft and related trades workers

Group 8: Plant and machine operators and assemblers

Stationary plant and related operators
Machine operators and assemblers
Drivers and mobile plant operators

Group 9: Elementary occupations

Sales and services elementary occupations
Agricultural, fishery and related labourers
Labourers in mining, construction, manufacturing and transport

Group 10: Armed forces

Armed forces

Group 11: Homemaker

Housewife/Househusband

Subject ID

☐☐ Centre # ☐☐☐ Community# ☐☐☐ Household # ☐☐ Subject #

Subject Initials ☐☐☐ F M L

10. Not applicable in South Africa

11a) Not applicable in South Africa

b) Please indicate which group best describes your main occupation.
(Please refer to facing page for definitions of groups and instruction manual for detailed definitions)

☐ Group 1 ☐ Group 2 ☐ Group 3 ☐ Group 4 ☐ Group 5
☐ Group 6 ☐ Group 7 ☐ Group 8 ☐ Group 9 ☐ Group 10 ☐ Group 11

c) Not applicable in South Africa

d) What is your main source of income? ______________________________

If occupation is group 11 (homemaker) go to question 13

12. Are you currently employed?

☐ No → (answer 12a - 12b) ☐ Yes → Go to #13

a) Are you retired/stopped work from your primary occupation due to old age? ☐ No ☐ Yes

b) Have you stopped working due to illness? ☐ No ☐ Yes

Subject ID

☐☐	☐☐☐	☐☐☐	☐☐
Centre #	*Community#*	*Household #*	*Subject #*

Subject Initials ☐☐☐ *F M L*

13. CURRENT DISABILITY:

		No	Yes
a)	Do you have any problems using your fingers to grasp or handle?	☐	☐
b)	Do you have any trouble walking about?	☐	☐
c)	Do you have any trouble bending down and picking up an object from the floor?	☐	☐
d)	Do you require a walking stick cane/walker to move about?	☐	☐
e)	Do you have any trouble reading or seeing the individual grains of rice/corn on your plate? (with glasses worn)	☐	☐
f)	Do you have trouble seeing a person from across the room? (12 feet/3.5 meters) (with glasses worn)	☐	☐
g)	Do you have trouble speaking and being understood?	☐	☐
h)	Do you have any trouble hearing what is said in a normal conversation?	☐	☐

Subject Medical History

14. Have you experienced any of the following in the last six months?

	No	Yes		No	Yes
a) Chest pain or tightness with usual activity	☐	☐	i) Vomiting	☐	☐
If Yes, → does the pain spread to the back, neck or inner border of arm	☐	☐	j) Loss of appetite	☐	☐
b) Breathlessness with usual activity	☐	☐	k) Painful or bleeding teeth/gums	☐	☐
c) Cough for at least 2 weeks	☐	☐	l) Jaundice	☐	☐
d) Any sputum while coughing	☐	☐	m) Burning while passing urine	☐	☐
e) Blood in sputum	☐	☐	n) Swelling of feet	☐	☐
f) Wheezing or whistling in the chest	☐	☐	o) Swelling of face	☐	☐
g) Early morning cough with chest tightness	☐	☐	p) Blood in urine	☐	☐
h) Loose stools/diarrhea for at least 3 days	☐	☐	q) Involuntary weight loss of > 3kg	☐	☐

15. Not applicable in South Africa

16a) Do you use glasses/spectacles/contact lenses at present? No ☐ Yes ☐

b) Do you use a hearing aid? No ☐ Yes ☐

Adult Questionnaire

Cancer Sites

1= Mouth
2= Esophagus
3= Stomach
4= Small intestine
5= Large intestine including rectum
6= Pancreas
7= Liver
8= Lung
9= Breast
10= Cervical/uterine/ovarian
11= Prostate
12= Head and neck
13= Other, specify

Subject ID

☐☐ Centre # ☐☐☐ Community# ☐☐☐ Household # ☐☐ Subject #

Subject Initials ☐☐☐ F M L

17. Have you ever been diagnosed with any of the following?(check all that apply)

	No	Yes	#of yrs since diagnosis
a) Diabetes	☐	☐	☐☐
b) Hypertension/ high blood pressure	☐	☐	☐☐
c) Stroke	☐	☐	☐☐
d) Angina/heart attack/ Coronary artery disease	☐	☐	☐☐
e) Heart failure	☐	☐	☐☐
f) Other heart disease	☐	☐	☐☐
h) Hepatitis/Jaundice	☐	☐	☐☐
g) Cancer	☐	☐	☐☐

Please refer to facing page for cancer sites ☐☐ *site* ______ *other, specify*

	No	Yes	#of yrs since diagnosis
i) COPD	☐	☐	☐☐
j) Asthma	☐	☐	☐☐
k) Tuberculosis	☐	☐	☐☐
l) Malaria	☐	☐	☐☐
m) Chagas	☐	☐	☐☐
n) HIV/AIDS (Not answered ☐)	☐	☐	☐☐

18. Have you been taking any medications regularly (ie. at least once per week) in the last month? ☐ No → **go to 19** ☐ Yes

a) If yes, for what conditions:

	No	Yes
Blood pressure	☐	☐
Cholesterol lowering drugs	☐	☐
Stroke	☐	☐
Diabetes	☐	☐
Asthma	☐	☐
Chinese medicine	☐	☐
Others	☐	☐ → If Yes, specify ______
Unknown	☐	☐

Adult Questionnaire

18b) If name of medication is unknown, please list as unknown.

Subject ID

☐☐	☐☐☐	☐☐☐	☐☐
Centre #	*Community#*	*Household #*	*Subject #*

Subject Initials ☐☐☐ *F M L*

18b) List all the medications you are <u>currently</u> consuming at least once a week for the last month?

i) ____________________ ii) ____________________

iii) ____________________ iv) ____________________

v) ____________________ vi) ____________________

vii) ____________________ viii) ____________________

<u>Men go to question #23</u>

<u>For Women Only (Questions 19 - 22)</u>

19. Are you currently pregnant ? ☐ No ☐ Yes → **Go to #21**

20. Do you still have periods? ☐ No → **(answer 20a)** ☐ Yes → **Go to #21**

a) How many years since you stopped menstruating? ☐☐ years

21. Have you ever used an oral/ injectable contraceptive? ☐ No ☐ Yes

22a) How many live children have you given birth to? ☐☐ Boys ☐☐ Girls

b) Did you breast feed any of your children? ☐ No ☐ Yes

Adult Questionnaire

23. Accidents and Injuries

Location of Injury
1= Factory/industrial place
2= Office
3= Agriculture field/farm
4= Home
5= Road
6= Sport/game e.g. track, court, field, etc.
7= Public building
8= Mine/quarry
9= Construction site e.g. building, road-works, etc.
10 = Other

Type of Injury
1= Burns
2= Scalds
3= Fractures
4= Muscle and ligament sprains/tears
5= Cuts and lacerations
6= Bruises and abrasions
7= Suffocation
8= Head injury (where person did not lose consciousness)
9= Head injury (where person lost consciousness for some time)

Subject ID

Centre #	Community#	Household #	Subject #

Subject Initials F M L

23. **During the past 12 months, have you had any injuries that were serious enough to limit your normal activities?** (check all that apply)

☐ No → **Go to #24** ☐ Yes → **(answer 23a - 23s)**

If yes, please provide details:

Please refer to facing page for Location and Type Codes

Cause of injury			Location	Type	Absence from work or usual activities (Days)
a) Motor vechicle accident (as a passenger)	☐ No	☐ Yes →			
b) Motor vehicle accident (as a pedestrian)	☐ No	☐ Yes →			
c) Struck by an object	☐ No	☐ Yes →			
d) Explosion	☐ No	☐ Yes →			
e) Natural/environmental factors (gales/cyclones/lightning, etc.)	☐ No	☐ Yes →			
f) Suffocation	☐ No	☐ Yes →			
g) Poisoning	☐ No	☐ Yes →			
h) Snake/scorpion bite	☐ No	☐ Yes →			
i) Fall	☐ No	☐ Yes →			
j) Fire/flames, resultant fumes	☐ No	☐ Yes →			
k) Physical assault (gun, kidnapping, etc.)/violent crime	☐ No	☐ Yes →			
l) Domestic violence (beaten by a family member)	☐ No	☐ Yes →			
m) Drowning/submersion	☐ No	☐ Yes →			
n) Hot or corrosive liquids/floods/substances	☐ No	☐ Yes →			
o) Crush injuries (boulders, building materials, etc.)	☐ No	☐ Yes →			
p) Accident caused by machinery	☐ No	☐ Yes →			
q) Attempted suicide	☐ No	☐ Yes →			
r) Armed conflict	☐ No	☐ Yes →			
s) Other(specify) ______________	☐ No	☐ Yes →			

Adult Questionnaire

Location of Fractures

1= Hip/pelvis
2= Thigh
3= Leg
4= Forearm
5= Wrist
6= Hand/finger
7= Vertebrae (back)
8= Other

Fractures: In situations where subjects are in a cast and cannot differentiate between ligament tear or fracture, include as fracture only if doctor confirmed it as a broken bone

25c) Tobacco: Regular use is defined as consuming at least one tobacco product per day.

Duration of use:

For those that have consumed tobacco for <1 year, please enter "0"

Subject ID

☐☐ Centre # ☐☐☐ Community# ☐☐☐ Household # ☐☐ Subject #

Subject Initials ☐☐☐ F M L

24. Have you ever fractured a bone? ☐ No (go to #25) ☐ Yes (if yes, answer a),b) and c)

a) Number of fractures ☐☐

b) Years since last fracture ☐☐ (yrs)

c) Bone (s) broken in the most recent fracture(if more than 3, list most severe sites)

Please refer to facing page for fracture locations

(location) If other, specify

☐ → ____________

☐ → ____________

☐ → ____________

Tobacco

25. Which best describes your history of tobacco use?

a) ☐ Formerly used tobacco products ☐ Currently use tobacco products ☐ **Never used tobacco products** → **Go to #26**

b) At what age did you start? ☐☐ yrs

c) Have you ever regularly used any of the following tobacco products? (check all that apply)

	**Average amount/day**		**Duration** (years)	**Past users only** **When Stopped** (years ago)	**If less than 1 yr** (months ago)
(i) Cigarettes (all kinds)	☐☐☐	number	☐☐	☐☐	☐☐
(ii) Beedies	☐☐☐	number	☐☐	☐☐	☐☐
(iii) Cigars	☐☐☐	number	☐☐	☐☐	☐☐
(iv) Pipes	☐☐☐	number	☐☐	☐☐	☐☐
(v) Sheesha/water pipe Hookah	☐☐☐	# of times	☐☐	☐☐	☐☐
(vi) Chewing tobacco	☐☐☐	# of times	☐☐	☐☐	☐☐
(vii) Snuff	☐☐☐	# of times	☐☐	☐☐	☐☐
(x) Other ________ *Specify*	☐☐☐		☐☐	☐☐	☐☐

Subject ID

☐☐ *Centre #* ☐☐☐ *Community#* ☐☐☐ *Household #* ☐☐ *Subject #*

Subject Initials ☐☐☐ *F M L*

<u>Question 26 to be answered by non-smokers and former smokers only</u>

26. **During the past 12 months, have you been regularly (at least once per week) exposed to other people's tobacco smoke?**
("Exposed" is defined as a minimum of 5 consecutive minutes, during which you inhale other people's smoke.)

☐ No ⟶ **Go to #27** ☐ Yes ⟶ Please answer questions **26a**

a) Over the past 12 months, what has been your typical exposure to other peoples smoke?
("Exposed" is defined as a minimum of 5 consecutive minutes, during which you inhale other peoples smoke)

Select <u>ONE</u> only

☐ 1-2 times/week ☐ 3-6 times/week ☐ at least once a day ☐ 2-3 times/day ☐ 4 or more times/day

27. Not applicable in South Africa

Adult Questionnaire

28c) Alcoholic Beverage: Regular use is defined as at least once a month.

Subject ID

☐☐ Centre # ☐☐☐ Community# ☐☐☐ Household # ☐☐ Subject #

Subject Initials ☐☐☐ F M L

28. Which best describes your history of alcohol use?

a) ☐ Formerly used alcohol products ☐ Currently use alcohol products ☐ **Never used alcohol products** → **Go to #29**

b) At what age did you start? ☐☐ yrs

c) What forms of alcohol have you regularly used? (check all that apply)

Form of Alcohol	Approx. size of one "drink"	Frequency Daily	Weekly	Monthly	Average # of drinks	Duration (years)	Past users only When Stopped (years ago)
(i) Spirits(rum,whisky, gin,vodka etc)	30ml	☐	☐	☐	☐☐	☐☐	☐☐
(ii) Wine	125ml	☐	☐	☐	☐☐	☐☐	☐☐
(vi) Beer	375ml	☐	☐	☐	☐☐	☐☐	☐☐
(vii) Country liquor/arrack/ sugar cane spirit	30ml	☐	☐	☐	☐☐	☐☐	☐☐

d) **At least once a month, do you consume >5 alcoholic drinks/day?** ☐ No → **Go to #29** ☐ Yes

↓ **If yes,(i,ii)**

i) How many times per month do you consume >5 alcoholic drinks in a day? ☐☐

ii) What is the average number of drinks that you consume each time? ☐☐

29 a) **During your longest or nocturnal sleep period, what time do you normally go to bed?** ☐☐:☐☐ (00:00-23:59)

b) **During your longest or nocturnal sleep period, what time do you normally wake up?** ☐☐:☐☐ (00:00-23:59)

c) **Do you usually take naps/siestas?** ☐ No ☐ Yes *Total nap duration* → ☐☐☐ mins

Adult Questionnaire

33. <u>Civic organization</u>: are defined as non-profit, voluntary organization societies, self help groups and clubs.

    <u>Religious organization</u>: are defined as different types of formal and informal groups set up on a religious basis.

Subject ID

☐☐ Centre # ☐☐☐ Community# ☐☐☐ Household # ☐☐ Subject #

Subject Initials ☐☐☐ F M L

30. Are you a member of any of the following:

How often do you participate in the activities of this group?

			Per Month	OR	Per Year
(i) Self help group, Co-operative, Social club, Sports club,	☐ No	☐ Yes →	☐☐		☐☐
(ii) Religious Group (e.g: church group, etc.)	☐ No	☐ Yes →	☐☐		☐☐
(iii) Other ________ *Specify*	☐ No	☐ Yes →	☐☐		☐☐

31. Please answer the following: (choose only one option for each)

	Strongly Disagree	Somewhat Disagree	Somewhat Agree	Strongly Agree
(i) People are generally honest and want to help others.	☐	☐	☐	☐
(ii) If I do nice things for someone, I can anticipate that they will respect me and treat me just as well as I treat them.	☐	☐	☐	☐

32a) The television, radio, newspaper or magazine advertisements help me decide to buy the type of: (choose only one option for each)

	Strongly Disagree	Somewhat Disagree	Somewhat Agree	Strongly Agree	Not Applicable
(i) Cooking oil	☐	☐	☐	☐	☐
(ii) Flour	☐	☐	☐	☐	☐
(iii) Rice/ Maize meal	☐	☐	☐	☐	☐

b) The television, radio, newspaper or magazine advertisements influence whether I buy: (choose only one option for each)

	Strongly Disagree	Somewhat Disagree	Somewhat Agree	Strongly Agree	Not Applicable
(i) Soft drinks	☐	☐	☐	☐	☐
(ii) Snacks	☐	☐	☐	☐	☐
(iii) Cigarettes	☐	☐	☐	☐	☐
(iv) Alcohol	☐	☐	☐	☐	☐

33. In a difficult situation, whose help can you count on from?(Please see facing page for definitions)

(i) Civic organizations: specify ________________

☐ none ☐ little ☐ moderate/average ☐ a great deal

(ii) Religious organizations: specify ________________

☐ none ☐ little ☐ moderate/average ☐ a great deal

Subject ID

☐☐ *Centre #* ☐☐☐ *Community#* ☐☐☐ *Household #* ☐☐ *Subject #*

Subject Initials ☐☐☐ *F M L*

34. Have you experienced any of the following events during the last 12 months?

	No response	No	Yes	
(i) Loss of job	☐	☐	☐	
(ii) Retirement	☐	☐	☐	
(iii) Loss of crop/business failure	☐	☐	☐	
(iv) Household break in	☐	☐	☐	
(v) Marital separation/divorce	☐	☐	☐	
(vi) Other major intra-family conflict	☐	☐	☐	→ Please specify ______
(vii) Major personal injury or illness	☐	☐	☐	
(viii) Violence	☐	☐	☐	
(ix) Armed conflict/war	☐	☐	☐	
(x) Death of a spouse	☐	☐	☐	
(xi) Death/major illness of another close family member	☐	☐	☐	
(xii) Other major stress	☐	☐	☐	→ Please specify ______
(xiii) Wedding of family member	☐	☐	☐	
(xiv) New job	☐	☐	☐	
(xv) Birth in the family	☐	☐	☐	
(xvi) Seperation from family	☐	☐	☐	
(xvii) Unavailability of food/ food insecurity	☐	☐	☐	

Subject ID

☐☐	☐☐☐	☐☐☐	☐☐
Centre #	*Community#*	*Household #*	*Subject #*

Subject Initials ☐☐☐
F M L

35. **Please answer the following:** (Choose only one option for each)

For the following question, stress is defined as feeling irritable or filled with anxiety, or as having sleeping difficulties as a result of conditions at work or at home.

	No response	Never Experienced Stress	Some Period of Stress	Several Periods of Stress	Permanent Stress
a) How often have you felt stress at work in the last 12 months? (Mark here if not applicable: i.e. no longer working ☐)	☐	☐	☐	☐	☐
b) How often have you felt stress at home in the last 12 months?	☐	☐	☐	☐	☐

36. What level of financial stress have you felt in the last 12 months?

☐ No response ☐ Little/none ☐ Moderate ☐ High/severe

37. **During the past twelve months, was there ever a time when you felt sad, blue, or depressed for two weeks or more in a row?**

☐ No ☐ Yes ⟶ **If yes, during those times, did you:**

	No response	No	Yes
a) Lose interest in most things like hobbies, work or activities that usually give you pleasure?	☐	☐	☐
b) Feel tired or low on energy?	☐	☐	☐
c) Gain or lose weight?	☐	☐	☐
d) Have more trouble falling asleep than you usually do?	☐	☐	☐
e) Have more trouble concentrating than usual?	☐	☐	☐
f) Think a lot about death (either your own, someone else's, or death in general)	☐	☐	☐
g) Feel down on yourself, no good or worthless?	☐	☐	☐

Subject ID

Centre #	*Community#*	*Household #*	*Subject #*

Subject Initials *F M L*

38. **Please answer the following:** (Choose only one option for each)

	Strongly Disagree	Somewhat Disagree	Somewhat Agree	Strongly Agree
a) I can do most of my regular shopping (food, household necessities, etc.) at stores within easy walking distance (less than 15 minutes) of my home.	☐	☐	☐	☐
b) Walking or bicycling in my neighbourhood is difficult because of the speed and/or amount of traffic.	☐	☐	☐	☐
c) My neighbourhood is generally free from pollution (litter, air pollution and noise pollution).	☐	☐	☐	☐
d) My neighbourhood streets are well lit at night.	☐	☐	☐	☐
e) I can see other people when I am walking in my neighbourhood.	☐	☐	☐	☐
f) I can speak to other people when I am walking in my neighbourhood.	☐	☐	☐	☐
g) There is a high crime rate in my neighbourhood.	☐	☐	☐	☐
h) There is a problem with unattended dogs in my neighbourhood.	☐	☐	☐	☐

Subject ID

☐☐	☐☐☐	☐☐☐	☐☐
Centre #	*Community#*	*Household #*	*Subject #*

Subject Initials ☐☐☐
F M L

38a) Please answer the following: (Please check all that apply)

i) Has your household been a victim of the following crime(s) in the last 12 months?

	No	Yes
1. Armed robbery	☐	☐
2. Violent attacks	☐	☐
3. Murder	☐	☐
4. Vehicle hijacking	☐	☐
5. House breaking	☐	☐
6. Theft	☐	☐
7. Rape	☐	☐
8. Women abuse eg. (beat,swear-words,sexual) please specify ________	☐	☐
9. Child abuse eg. (burn,swear-words,rejection) please specify ________	☐	☐
10. Child sexual abuse	☐	☐
11. Other, please specify ________	☐	☐

ii) Do you think that crime in your area has increased in the past 5 years? ☐ No ☐ Yes

if yes, which of the following crime(s)?

- ☐ Armed robbery
- ☐ Violent attacks
- ☐ Murder
- ☐ Vehicle hijacking
- ☐ House breaking
- ☐ Theft
- ☐ Rape
- ☐ Women abuse
- ☐ Child abuse
- ☐ Child sexual abuse
- ☐ Other, please specify ________

Subject ID

☐☐ Centre # ☐☐☐ Community# ☐☐☐ Household # ☐☐ Subject #

Subject Initials ☐☐☐ F M L

38b) Questions on HIV:

i) Do you know people who have HIV/AIDS? ☐ No ☐ Yes

if yes, which of these people: (please mark all that apply)

- ☐ Your children
- ☐ Your grandchildren
- ☐ Your spouse
- ☐ Your family members
- ☐ Your friends
- ☐ People in the community

ii) What would you consider the mean age of the people who are ill/have died of HIV/AIDS?

☐ Younger than 10 years ☐ Between 11-20 years ☐ Between 21-30 years

☐ Between 31-40 years ☐ Between 41-50 years ☐ Over 50 years

iii) If someone in your household is HIV positive, who is the primary caregiver?

- ☐ Spouse
- ☐ Parents
- ☐ Family member
- ☐ Child.children
- ☐ Friends
- ☐ Volunteer

38c) Do you care for any orphans in your family? ☐ No ☐ Yes

Adult Questionnaire

40b) Health History:

Cancer Sites

1= Mouth
2= Esophagus
3= Stomach
4= Small intestine
5= Large intestine including rectum
6= Pancreas
7= Liver
8= Lung
9= Breast
10= Cervical/uterine/ovarian
11= Prostate
12= Head and neck
13= Other, specify

Subject ID

Centre # | Community# | Household # | Subject #

Subject Initials F M L

39. How long would it take you to get from your house to the nearest facility <u>if you walked</u>?

	Minutes	Don't know		Minutes	Don't know
i) grocery/convenience store			iv) video store		
ii) bank			v) non-fast food restaurant		
iii) post office			vi) fast food restaurant		

40a) Total number of siblings

b) Health History: Complete for all parents and siblings, alive or dead

	Father			Mother			Siblings			# of siblings with the condition
	Unknown	No	Yes	Unknown	No	Yes	Unknown	No	Yes	If yes →
Diabetes										
Coronary Heart Disease										
High Blood Pressure										
Stroke										
Cancer (Please refer to facing page for cancer sites)										

→ if Yes, indicate site

Other, Specify | Other, Specify | Other, Specify

Adult Questionnaire

If subject refuses to provide any of the measures, enter a value of "0" into each of the boxes for that question

For more detailed instructions please refer to the instruction manual

Subject ID

Centre # | Community# | Household # | Subject #

Subject Initials F M L

41. **Physical Measurements**

a) Sitting Right arm blood pressure
#1 ___ ___ mmHg
Systolic Diastolic
#2 ___ ___ mmHg
Systolic Diastolic

b) Heart Rate
#1 ___ beats/min
#2 ___ beats/min

c) Waist
#1 ___ . _ cm → ☐ minimal/no clothing ☐ full clothing
#2 ___ . _ cm

d) Weight ___ . _ kg → ☐ minimal/no clothing ☐ full clothing

e) Hip
#1 ___ . _ cm → ☐ minimal/no clothing ☐ full clothing
#2 ___ . _ cm

f) Height ___ . _ cm (without shoes)

42a) Circumference of mid upper right arm: __ . _ cm

b) Circumference of right calf: __ . _ cm

c) Head Circumference: ___ . _ cm

d) Upper flexed arm circumference __ . _ cm

43a) Right arm triceps skinfold:
#1 ___ . _ mm
#2 ___ . _ mm
#3 ___ . _ mm

b) Right calf skinfold:
#1 ___ . _ mm
#2 ___ . _ mm
#3 ___ . _ mm

Subject ID

Centre # Community# Household # Subject #

Subject Initials F M L

c) Biceps skinfold #1 ___ . _ mm #2 ___ . _ mm #3 ___ _ mm

d) Subscapular skinfold #1 ___ . _ mm #2 ___ . _ mm #3 ___ _ mm

e) Supra spinal skinfolds #1 ___ . _ mm #2 ___ . _ mm #3 ___ _ mm

44 a) Humerous breadth __ . _ cm

b) Femur breadth __ . _ cm

45. Grip Strength (Maximal contraction):

a) Non-dominant hand: #1 __ kg. #2 __ kg. #3 __ kg.

b) Dominant hand: #1 __ kg. #2 __ kg. #3 __ kg.

Adult Questionnaire

If subject refuses to provide any of the measures, enter a value of "0" into each of the boxes for that question

For more detailed instructions please refer to the instruction manual

46. Spirometry:

American Thoracic Society criteria for acceptable spirograms:
Spirograms are acceptable if they are free from:

1. Cough during exhalation
2. Early termination or cut-off
3. Variable effort
4. Leaks
5. Obstructed mouth piece

Subject ID

☐☐ *Centre #* ☐☐☐ *Community#* ☐☐☐ *Household #* ☐☐ *Subject #*

Subject Initials ☐☐☐ *F M L*

46. Spirometry:

a) **FEV1 (Litre):** #1 ☐.☐☐ #2 ☐.☐☐ #3 ☐.☐☐

b) Does FEV1 obtained meet ATS criteria?

☐ No ⟶ (answer (i) to (iii) ☐ Yes ⟶ Go to **c)**

Reasons for not meeting the ATS criteria: (check all that apply)

i) Cough ☐

ii) Values not within 0.2L of each other ☐

iii) Less than 3 values ☐

c) **FVC (Litre):** #1 ☐.☐☐ #2 ☐.☐☐ #3 ☐.☐☐

d) Does FVC obtained meet ATS criteria?

☐ No ⟶ (answer (i) to (iii) ☐ Yes ⟶ Go to **e)**

Reasons for not meeting the ATS criteria: (check all that apply)

i) Cough ☐

ii) Values not within 0.2L of each other ☐

iii) Less than 3 values ☐

e) **PEFR (Litre/min):** #1 ☐☐☐ #2 ☐☐☐ #3 ☐☐☐

f) Does PEFR obtained meet ATS criteria?

☐ No ⟶ (answer (i) to (ii) ☐ Yes ⟶ Go to **Q#47**

Reasons for not meeting the ATS criteria: (check all that apply)

i) Cough ☐

ii) Less than 3 values ☐

Subject ID

☐☐ Centre # ☐☐☐ Community# ☐☐☐ Household # ☐☐ Subject #

Subject Initials ☐☐☐ F M L

47. **Not applicable in South Africa**

48. **ECG obtained?** No ☐ → **Go to #49** Yes ☐

a) 2 0 _ _ (year) _ _ (month) _ _ (day) → Place ECG :File Label Here

b) **Please print ECG label #:** ☐☐☐☐☐☐

49 a) **Blood sample obtained?** No ☐ → **Go to #50** Yes ☐

b) ☐ Fasting sample ☐ Non-fasting sample

c) 2 0 _ _ (year) _ _ (month) _ _ (day) **Time** ☐☐ : ☐☐ (00:00-23:59) → **Hours since any food/beverage consumed (excluding water)** ☐☐

d) **Please print Blood label #:** ☐☐☐☐☐☐ Place Blood label here

50 a) **Urine sample obtained?** No ☐ → **Go to #51** Yes ☐

b) ☐ Fasting sample ☐ Non-fasting sample

c) **Please print Urine label #:** ☐☐☐☐☐☐ Place Urine label here

51. **Name of Interviewer:** *(please print)* ______ *First Initial* ______ *Last Name*

Interviewer Code: ☐☐☐
