# Supplementary material for: Prognostic values of modifiable risk factors for cardiovascular events in South African health promotion
Source: PLoS One. 2022 Aug 10;17(8):e0271169. doi: 10.1371/journal.pone.0271169 (PMC9365156; doi:10.1371/journal.pone.0271169)
Supplement: S2 File — (PDF) [file pone.0271169.s002.pdf]

YUNIBESITI YA BOKONE-BOPHIRIMA
NORTH WEST UNIVERSITY
NOORDWES UNIVERSITEIT

PURE-SA Project (Prospective Urban and Rural Epidemiology)

Physical activity questionnaire

Date: Place: Interviewer:

The information on this questionnaire is confidential

1.	Subject number						(1-4)
2.	Gender			Male	1	Female 2	(5)
3.	What is your main occupation?...............................						
	Low level: office work, housework, scholar					1	
	Middle level: factory work, carpentry, farming, hospital nurse, plumber					2	(6)
	High level ("sweat work"): construction work, digging, manual labour					3	
4.	At work I sit	1. never	2. seldom	3. sometimes	4.often	5.always	(7)
5.	At work I stand	1. never	2. seldom	3. sometimes	4.often	5.always	(8)
6.	At work I walk	1. never	2. seldom	3. sometimes	4.often	5.always	(9)
7.	At work I lift heavy loads	1. never	2. seldom	3. sometimes	4.often	5.always	(10)
8.	At work I am tired	1.never	2.seldom	3. sometimes	4.often	5.always	(11)
9.	At work I sweat	1.never	2.seldom	3. sometimes	4.often	5.always	(12)
10.	If you work away from home, how do you get to work/school?				walk	1	
					cycle	2	(13)
					car/taxi	3	
11.	How long does it take you to walk/cycle to work/school? (or to the taxi rank/ bus stop/ train station)				0-15 min	1	
					16-30 min	2	
					31-60 min	3	
					1-2 hours	4	(14)
12.	If you walk or cycle to work/school, what is your usual pace? (or to taxi rank/bus stop/ train station)				casual strolling	1	
					fairly brisk	2	
					brisk/fast	3	(15)
13.	Do you climb stairs often?				yes	1	
					no	2	(16)
14.	If yes, how many flights of stairs do you climb each day? (1 flight = 10 steps)						(17)
15.	How many days per week do you climb steps?						(18)
16.	Do you play sport?				yes	1	
					no	2	(19)
17.	Which sport do you play most frequently?				low level: bowling, golf, billiards	1	0.76*1
					middle level: tennis, athletics, cycling	2	1.26
					high level: soccer, rugby, netball, boxing	3	1.76(20)
18.	How many hours per week do you practice?<1/ 1-2/ 2-3/ 3-4/ >4 (Write appropriate code in space) 0.5, 1.5, 2.5, 3.5, 4.5*2					.	(21-23)
19.	How many months per year? <1/ 1-3/ 4-6/ 7-9/ >9 (Write appropriate code in space) 0.04, 0.17, 0.42, 0.67, 0.92*3					.	(24-26)

*1 intensity code of sport, *2 time code for sport, *3 proportion of year

20.	If you play a second sport, which is it?	low level: bowling, golf, billiards				1	0.76*[1]
		middle level: tennis, athletics, cycling				2	1.26
		high level: soccer, rugby, netball, boxing				3	1.76(27)
21.	How many hours per week do you practice?	<1/ 1-2/ 2-3/ 3-4/ >4<br>0.5, 1.5, 2.5, 3.5, 4.5*[2]				.	(28-30)
22.	How many months per year?	<1/ 1-3/ 4-6/ 7-9/ >9<br>0.04, 0.17, 0.42, 0.67, 0.92*[3]				.	(31-33)
23.	During leisure time I watch TV/ do sitting activities (read, needle-work, play cards)	1.never	2.sel-dom	3.some -times	4.often	5.al-ways	(34)
24.	During leisure time I walk/ do standing activities (gardening, housework)	1.never	2.sel-dom	3.some -times	4.often	5.al-ways	(35)
25.	Other leisure-time activities:.................... (leisure-time = time off from work/. school)		2.sel-dom	3.some -times	4.often	5.al-ways	(36)
